# Implementation of High Resolution Whole Genome Array CGH in the Prenatal Clinical Setting: Advantages, Challenges, and Review of the Literature

**DOI:** 10.1155/2013/346762

**Published:** 2013-03-04

**Authors:** Paola Evangelidou, Angelos Alexandrou, Maria Moutafi, Marios Ioannides, Pavlos Antoniou, George Koumbaris, Ioannis Kallikas, Voula Velissariou, Carolina Sismani, Philippos C. Patsalis

**Affiliations:** ^1^Department of Cytogenetics and Genomics, The Cyprus Institute of Neurology and Genetics, 2370 Nicosia, Cyprus; ^2^Professor Patsalis Research Team, The Cyprus Institute of Neurology and Genetics, 2370 Nicosia, Cyprus; ^3^Ultrasound and Fetal Medicine Centre, 2025 Nicosia, Cyprus; ^4^Department of Genetics and Molecular Biology, Gynecological, and Children's Hospital, Mitera Maternity, 15123 Athens, Cyprus

## Abstract

Array Comparative Genomic Hybridization analysis is replacing postnatal chromosomal analysis in cases of intellectual disabilities, and it has been postulated that it might also become the first-tier test in prenatal diagnosis. In this study, array CGH was applied in 64 prenatal samples with whole genome oligonucleotide arrays (BlueGnome, Ltd.) on DNA extracted from chorionic villi, amniotic fluid, foetal blood, and skin samples. Results were confirmed with Fluorescence In Situ Hybridization or Real-Time PCR. Fifty-three cases had normal karyotype and abnormal ultrasound findings, and seven samples had balanced rearrangements, five of which also had ultrasound findings. The value of array CGH in the characterization of previously known aberrations in five samples is also presented. Seventeen out of 64 samples carried copy number alterations giving a detection rate of 26.5%. Ten of these represent benign or variables of unknown significance, giving a diagnostic capacity of the method to be 10.9%. If karyotype is performed the additional diagnostic capacity of the method is 5.1% (3/59). This study indicates the ability of array CGH to identify chromosomal abnormalities which cannot be detected during routine prenatal cytogenetic analysis, therefore increasing the overall detection rate. In addition a thorough review of the literature is presented.

## 1. Introduction 

Since the 1970s that chromosomal analysis became available in prenatal diagnosis; it has proven to be a robust technique in detecting the majority of chromosomal abnormalities. With the use of amniocytes, starting in the second trimester of pregnancy [1], as well as cells isolated from chorionic villus samples in the first trimester of pregnancy [2], it was demonstrated that foetal material could be cultured to obtain sufficient metaphase cells to determine the karyotype of the foetus. These methods have been used extensively until today with many improvements over the years. A full karyotype analysed from either cultured amniocytes or chorionic villus samples can be obtained within 10 to 21 days. Furthermore chromosomal analysis can detect aneuploidy, structural rearrangements, and deletions/duplications of at least 3–10 Mb. Rapid aneuploidy tests being offered today like MLPA (Multiplex Ligation-Dependent Probe Amplification), QF PCR (Quantitative Fluorescent Polymerase Chain Reaction) are high throughput and provide rapid aneuploidy detection for certain chromosomes. They cannot, however, replace chromosomal analysis in all cases requiring invasive prenatal diagnosis, as there is a residual risk of 0.9% for a clinically significant chromosomal abnormality for all indications of invasive prenatal diagnosis [3]. As in the majority of cases with ultrasound abnormalities the karyotype in the foetus is normal, thus demonstrating the need for additional diagnostic tests with higher diagnostic capacity [4].

Array CGH is a high throughput method which can be applied and detect copy number changes to a resolution of even as low as 1 Kb. Genome-wide arrays are rapidly replacing conventional karyotyping in postnatal diagnostics, as they are increasingly performed for the evaluation of individuals with birth defects, dysmorphic features, and mental retardation. ISCA (International Standard Cytogenomic Array) Consortium [5] supports the use of array CGH as a first-line test and suggests reserving chromosomal G-banding analysis for specific cases like patients with obvious chromosomal syndromes such as Down syndrome and family history of chromosomal rearrangements.

Its introduction, however, in prenatal diagnosis is still limited but will definitely increase in the near future. Many groups have demonstrated that by applying array CGH in prenatal diagnosis in conjunction with chromosomal analysis, there was an additional detection of clinically significant genomic imbalances [5–9], proving its usefulness, as well as its limitations, in using this technique in prenatal diagnosis. The question remains though as to whether it can be fully integrated in prenatal diagnosis, solely or in conjunction with other assays, and replace conventional cytogenetics.

There are, however, several issues that need to be addressed before implementing array CGH in prenatal diagnosis such as (1) for which pregnancies array CGH should be carried out, whether for all pregnancies or for pregnancies with ultrasound abnormalities, (2) which array platform to use, (3) the need to set the appropriate calling criteria, (4) which confirmatory methods to use for the array CGH findings, and (5) pretest counselling.

Pretest counselling is especially important in the prenatal setting, and it should be carried out to inform parents of the possibility of the fortuitous discovery of a copy number variation (CNV) unrelated to the phenotype during array CGH analysis. It should be explained to the parents that there may be asymptomatic/presymptomatic results with array CGH analysis, and they should be allowed to decide whether they wish to be informed of these findings or not [10].

In the current study, we present our experience of using whole genome oligonucleotide array CGH during prenatal diagnosis in cases with a normal karyotype with abnormal ultrasound findings or an apparently balanced structural aberration and provide a summary of our results; in addition we present the value of array CGH in the characterization of previously known aberrations. The role of whole genome oligonucleotide array CGH in prenatal diagnosis will be further evaluated in an attempt to gain more insight on its use in the prenatal setting.

## 2. Materials and Methods

### 2.1. Patients and Samples

Samples included in this study were received between May 2010, and October 2012 for prenatal diagnosis using G-banded karyotype and whole genome array CGH methodology. Among the 1414 prenatal samples received within the above period in 65 cases both chromosomal and array CGH analyses were carried out. Included in this cohort of patients were 42 amniotic fluid samples, 20 chorionic villus samples, 2 foetal blood samples, and 1 skin sample. Gestational age varied from 12.2 to 33 weeks. Ultrasound screening was carried out during the first trimester of pregnancies, and the findings include increased nuchal translucency, hypoplastic nasal bone, talipes, intrauterine growth retardation, hydronephrosis, choroid plexus cyst, tetralogy of fallot, hydrops, cardiac anomalies, ventriculomegaly, micrognathia, and skeletal abnormalities of the extremities. 

These samples were further subcategorized into 5 categories (A–E) according to the chromosomal analysis results and the presence or absence of ultrasound findings (Table 1). 

### 2.2. Conventional Cytogenetics and FISH Analyses

Conventional cytogenetic G-Banding analysis was carried out on all samples included in this study (CV, amniotic fluid, foetal blood, and skin) using standard cytogenetic methodologies [11]. Fluorescence In Situ Hybridization (FISH) was performed, where needed, using commercially available probes according to the manufacturer's protocol (VYSIS Co., Downers Grove, IL, and Cytocell, Co., UK).

### 2.3. Microarray Comparative Genomic Hybridization (Array CGH)

DNA was extracted from CV/AF/Skin uncultured cells and from uncultured foetal blood using the Qiagen Mini and Midi Kits, respectively, according to the manufacturer's protocol (Qiagen, Valencia, CA), and concentration and purity of the extracted DNA were measured with the NanoDrop spectrophotometer (NanoDrop Technologies, Inc.). Following DNA extraction, the test and reference DNA of the same gender were cohybridized to the array of choice, as previously described [12]. Briefly, 500 ng of patient and reference DNA were labelled by random priming using Bio Prime labelling kit (Invitrogen, Carlsbad, CA, USA) with Cyanine 3 and Cyanine 5 (Amersham Biosciences, UK) fluorescent dyes, respectively. Pooled genomic DNA from peripheral blood leukocytes of phenotypically normal males or females from Promega (Promega, Madison, WI, USA) was used as reference. DNA was then hybridized on the arrays (CytoChip, BlueGnome Ltd., UK) using an automated slide processor (HS 4800, Tecan Inc., Mannedorf, Switzerland). Array images were then acquired using an Agilent laser scanner G2565B, and image files were quantified using Agilent's feature extraction software (V9.5.3.1) and analysed with the BlueFuse for microarrays software package (BlueGnome, Ltd., UK). In the current study two different oligonucleotide arrays were used with 105,000 or 180,000 probes (BlueGnome, Ltd., UK). These arrays can detect copy number changes >50 kb in 138 targeted regions (microdeletion/duplication loci) and >150 kb in the remainder of the genome. CytoChip ISCA arrays report the gene content of over 500 recognized disease regions, while they have genome-wide coverage, including subtelomeres and pericentromeres, and support the detection of imbalances as small as 60 Kb.

### 2.4. Array Data and Confirmatory Analysis

Array data was analysed using BlueFuse software analysis (BlueGnome Ltd., UK), and the reporting threshold was set at 200 kb. Called imbalances were further aligned with the in-house database as well as to known aberrations listed in publically available databases, such as the DECIPHER (Database of Chromosomal Imbalance and Phenotype in Humans using Ensembl Resources http://decipher.sanger.ac.uk) and the Database of Genomic Variants (DGV, http://projects.tcag.ca/variation/) using NCBI136/hg18 UCSC or GRCh37/hg19 assemblies. Parental samples were analysed by array CGH only when needed. All copy number variations found were confirmed by FISH or Real-Time Polymerase Chain Reaction (RT-PCR) which were performed using previously described standard procedures [13, 14].

For a copy number change (CNC) to be considered as clinically significant/pathogenic the following criteria were applied:

1. the aberration had to be *de novo* or inherited from an affected parent;
2. the region contained genes and/or overlapped with a known syndrome or with a DECIPHER entry;
3. the region was not listed as polymorphic in DGV;
4. it was not previously found in the in-house database.

If an aberration met criteria 2 and 3 but was found in a normal parent and was not previously reported as a recurrent syndrome with variable phenotype due to incomplete penetrance, it was classified as a CNV of unclear significance.

All prospective parents were offered genetic counselling by the referring clinician and consented prior to the testing. 

## 3. Results and Discussion

### 3.1. Findings

A total of 65 samples/cases were included in this study out of which 40 and 25 were investigated using 105 K and 180 K oligonucleotide arrays, respectively. Out of the 65, one sample gave inconclusive results (failure rate 1/65, 1.53%). Consequently a total of 64 cases will be presented in this study.

A total of seventeen cases (17/64, 26.5%) with CNVs were determined by array CGH analysis, and the findings are listed in Table 2. Four out of the seventeen cases with CNVs detected in array CGH were abnormalities previously detected by other methods (G-banding and MLPA). Thirteen out of the seventeen CNVs detected were from pregnancies with a normal karyotype and ultrasound abnormalities out of which three (Cases 12, 34, and 38, 3/64, 4.7%) were pathogenic, while the remaining ten (10/64, 15.6%) were initially categorized as variables of unknown significance (VOUS). Following parental analysis seven out of the ten VOUS were determined to represent familial CNVs which were unrelated to the reason for referral. For three out of ten of those VOUS (Cases 52, 61, and 63) parental investigation is still on going. The diagnostic capacity of array CGH in the current cohort of prenatal cases is 10.93% (7/64) for clinically significant changes. From a total of five cases with abnormal findings previously identified by other methods (Groups D and E in Table 1), the aberration was confirmed and further characterized by array CGH in four cases (Cases 5, 9, 29, and 31). In one case, Case 7, in which chromosomal analysis determined an abnormal mosaic female karyotype with a supernumerary marker chromosome, array CGH, failed to determine the origin of the marker chromosome, suggesting that it most probably did not contain any euchromatic material.

### 3.2. Selected Case Presentations

#### 3.2.1. Pathogenic *De Novo *CNVs

**Figure 1:**
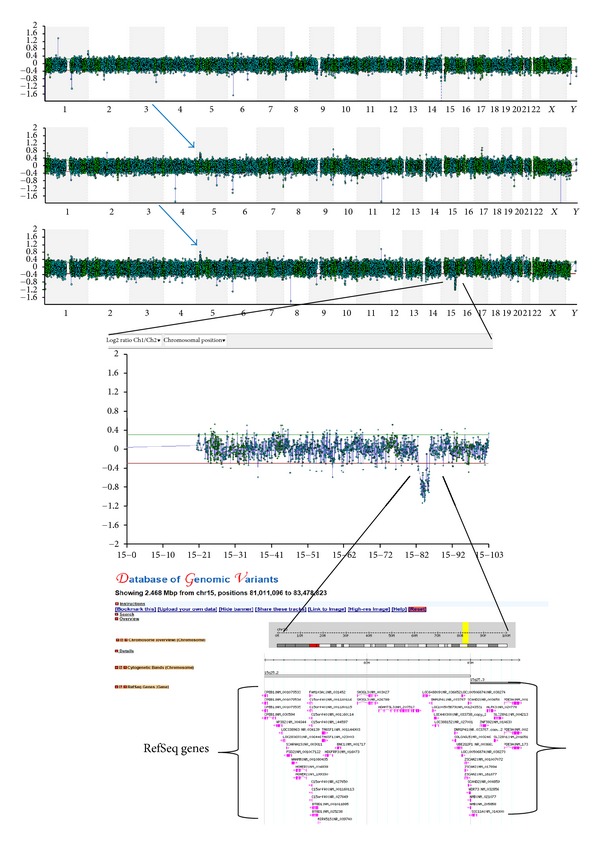
Case 12 showing a copy number gain on the short arm of chromosome 5 inherited from the healthy father and a *de novo* copy number loss on the long arm of chromosome 15. Representation of the chromosomal and genomic location region on chromosome 15 that has the copy number change in the Database of Genomic Variants. A loss of 2.4 Mb in size, which encompasses several RefSeq genes (shown in brackets); the region is not covered by any CNVs determining that it is not polymorphic.

Case 12 was a CVS sample from an 18-week pregnancy which was referred, initially, for chromosomal analysis, due to increased nuchal translucency. QF PCR analysis was carried out and revealed normal results. The sample was also treated as usually to establish cultures for chromosomal analysis, but after 14 days in culture there were no signs of growth. After obtaining consent from the patient and the physician, array CGH was carried out using 105 K oligonucleotide array on both the foetus and the parents. Array CGH revealed a duplication of 2.1 Mb in size on the short arm of chromosome 5, inherited from the healthy father, and a *de novo* deletion of 2.4 Mb in size on the long arm of chromosome 15 (Figure 1). The duplication on chromosome 5 was classified as likely benign, as it was inherited from the normal father, consequently stressing the necessity of confirming the presence/absence of CNVs in the parents to further categorize them. The deletion on chromosome 15 was reported as likely pathogenic, as it was relatively large in size, and it was *de novo*; the deleted region contained many genes and was not listed as polymorphic in the publicly available databases. Such single segmental imbalance even though it was determined by array CGH to be *de novo*, it could be the consequence of the unbalanced transmission of a derivative chromosome involved in an insertional balanced translocation (IT) in the parents [15]. Nowakowska et al. demonstrated that ITs underlie ~2.1% of apparently *de novo *interstitial CNVs. Such information may not be important to further evaluate the risk for the current foetus, but it is important for the accurate estimation of the recurrence risk to family members. Therefore chromosome visualization after microarray analysis is essential for delineating the rearrangement and assessing for further potential imbalance (in the immediate or even in the extended family). In the current case chromosomal analysis carried out in the parents did not detect an insertional translocation.

The deletion, however, was rather small in size for chromosomal analysis to detect (2.5 Mb); therefore FISH analysis would have been necessary to visualize exactly the nature of the imbalance. If FISH analysis cannot be performed in time for the prenatal case, a disclaimer should be written on the report regarding this point.

It is important to point out that in the current case, had chromosomal analysis been carried, out this aberration would have been missed.

#### 3.2.2. Pathogenic Familial CNVs

**Figure 2:**
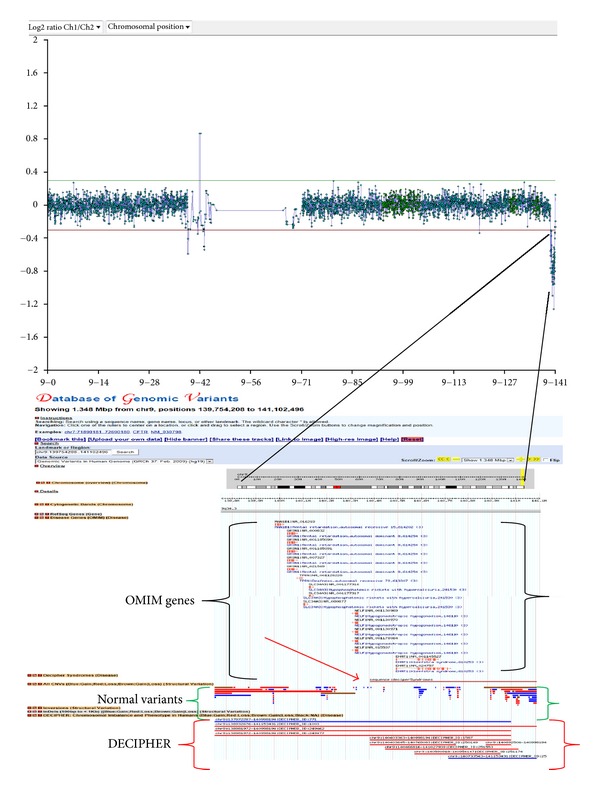
Case 34 showing a copy number loss on the long arm terminal of chromosome 9. Representation of the chromosomal and genomic location region on chromosome 9 that has the copy number change in the Database of Genomic Variants. A loss of 1.35 Mb in size which encompasses several OMIM genes (shown in brackets) and overlaps with a DECIPHER syndrome (the 9q microdeletion syndrome- shown by the red arrow). The area is not covered by a significant number of CNVs determining that it is not polymorphic.

**Figure 3:**
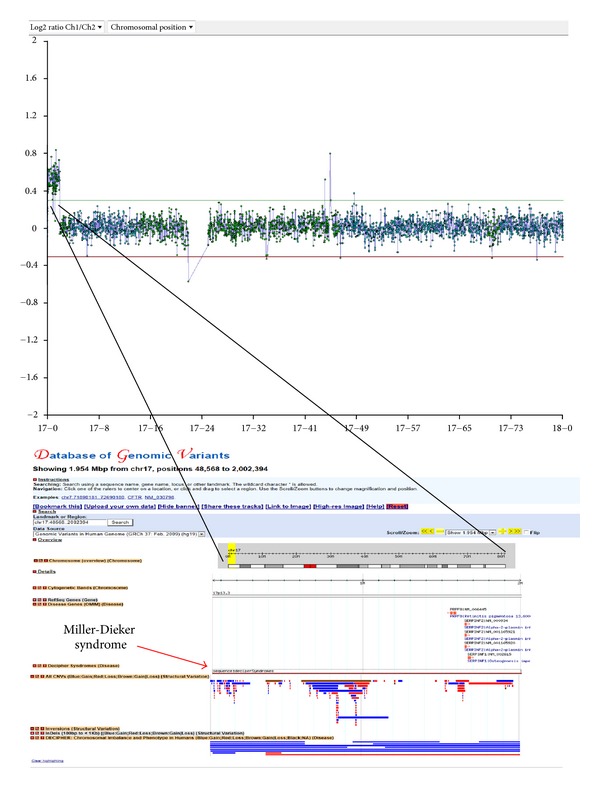
Case 34 showing a copy number gain on the short arm of chromosome 17. Representation of the chromosomal and genomic location region on chromosome 17 that has the copy number change in the Database of Genomic Variants. A gain of 1.95 Mb in size which encompasses some OMIM genes and overlaps with a DECIPHER syndrome (the Miller-Dieker syndrome—shown by the red arrow). The area is not covered by a significant number of CNVs determining that it is not polymorphic.

**Figure 4:**
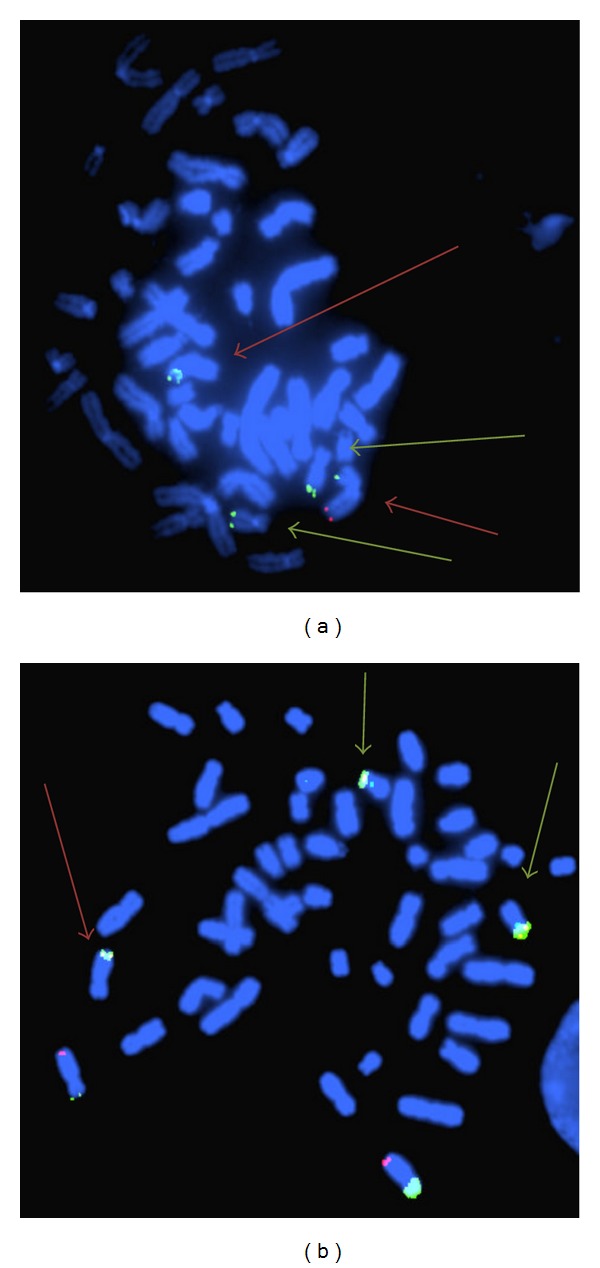
FISH analysis showing the confirmation of the unbalanced translocation in Case 34, using subtelomeric probes for chromosomes 9 and 17. Chromosome 8p and q probes (b) are also included in the probe mixture used (VYSIS, ToTelVysion probes). (a) Probes used: subtelomeric 9p and 17q, the top red arrow points at the derivative chromosome 9 (showing the deletion of 9q) and the green arrows point at chromosome 17. (b) Probes used: subtelomeric 17p, 8p and 8q, the red arrow points at the derivative chromosome 9 (showing the duplication of 17p) the green arrows point at chromosome17p.

Case 34, a 12-week pregnancy, was referred for chromosomal analysis and array CGH due to increased nuchal translucency (7.1 mm). Chromosomal analysis was normal (46,XY), but array CGH revealed double segmental imbalance which is usually an indication for the presence of an unbalanced translocation. Array CGH carried out with 105 K oligonucleotide array showed a terminal deletion on the long arm of chromosome 9, approximately 1.35 Mb in size, and a terminal duplication on the short arm of chromosome 17, approximately 1.95 Mb in size (Figures 2 and 3). FISH analysis, using subtelomeric specific probes for chromosomes 9 and 17, was then performed which confirmed the array CGH results and determined the presence of an unbalanced translocation (Figure 4). As expected, retrospective analysis of the foetus's karyotype could not detect any of the abnormalities, since the imbalances (1.35 Mb and 1.95 Mb) were beyond the resolution of the karyotype. Chromosomal and FISH analyses carried out in the parents revealed the presence of a submicroscopic apparently balanced translocation in the mother between the long-arm terminus of chromosome 9 and the short-arm terminus of chromosome 17. The subtelomeric 9 deletion found in the foetus includes many genes, several of which are OMIM genes. In addition, the duplicated region on chromosome 17 contained many genes including two OMIM genes and partially overlapped with the Miller-Dieker syndrome region. The couple went through counselling for further explanation of the implications of the findings for the current pregnancy, as well as for future pregnancies; the couple was elected to terminate the pregnancy.

The usefulness of the additional information array CGH provided in the diagnosis in this case is obvious; without it the copy number change would have remained undetected. Furthermore, the information acquired from this case will be used from the family for the better management of their pregnancies in the future. After careful evaluation of this couple's reproductive and medical history, it was revealed that they had a previous pregnancy (Case 38) which was terminated due to multiple severe ultrasound findings (tetralogy of Fallot, talipes, and other). In addition the couple also had an affected child. Both the previous pregnancy, and the child were previously karyotyped by our laboratory, and the results were normal. As expected, retrospective G-banding analysis of both the child and the previous pregnancy did not detect the abnormalities, and the parents consented to perform array CGH on stored genetic material from their previous pregnancy and their affected child. Array CGH analysis revealed related findings to the current case and contributed to the diagnosis for their affected child who had the same unbalanced karyotype as the analysed foetus. The importance of having the pedigree of a family being investigated is paramount as shown in this case. Had the parents informed the clinicians during the previous pregnancy that they already had an affected child; the management of the first pregnancy might have been different. The first pregnancy was investigated by chromosomal analysis on amniotic fluid sample on the 16th week and revealed normal karyotype. It was terminated based on the ultrasound findings despite the fact that the karyotype was apparently normal. Had the parents known at the time that their born child had a chromosomal abnormality which was inherited from the mother; they would have opted for an earlier prenatal diagnosis on their first pregnancy perhaps by chorionic villus sampling. This would have lessened their anxiety.

#### 3.2.3. Likely Benign CNVs/VOUS

The importance of carrying out confirmatory tests to the parents as well as the foetuses can also be seen in two other prenatal cases; CNVs found in the foetuses were classified as benign, after parental testing, as they were also present in healthy parents. Case 36, a 12-week pregnancy, was referred for chromosomal and array CGH analyses because of increased nuchal translucency. Array CGH analysis revealed a duplication of 0.5 Mb in size on the long arm of chromosome 7 which was classified to be benign, as it was also present in the healthy mother. Case 42, a 25-week pregnancy was referred for chromosomal analysis due to ultrasound findings (artrogryposis). Array CGH analysis revealed a duplication of 0.38 Mb in size on the short arm of chromosome 10 and a deletion of 0.32 Mb in size on the long arm of chromosome 15. Array CGH analyses carried out in the parents determined that the duplication was of paternal origin and the deletion was of maternal origin, determining that both CNVs were likely benign as each one was present in each one of the healthy parents. In Cases 52, 61, and 63 the CNVs found are considered variable of unknown significance (VOUS) as the abnormality still needs to be investigated through parental testing in order to determine if they represent clinically significant or benign CNVs.

It has to be pointed out that in the previous two cases array CGH analyses were carried out in the parents after extensive review of the publicly available databases (DGV, DECIPHER) as well as our own dataset. These databases did not show the CNVs found in these two cases to be common variants and that is why parental array CGH was subsequently carried out and showed that those CNVs were specific to that family.

### 3.3. Characterization of Previously Known Aberrations

Array CGH was able to characterize previously known abnormalities in four out of five cases. In three cases with marker chromosomes it confirmed the presence of additional genomic material and determined its size (Cases 5 and 29), but failed to confirm copy number gain in one case. Furthermore, array CGH delineated a deletion on the long arm of chromosome 7, in Case 9, which was identified by chromosomal analysis. The deletion was clinically significant, and it was determined to be approximately 6.3 Mb in size.

### 3.4. Array CGH Detection Rate in Prenatal Diagnosis

Many groups (Table 3) have demonstrated that by applying array CGH there was an additional detection of clinically significant genomic imbalances of approximately 3.6% (average from all studies) when the karyotype was normal, regardless of the indication of the referral for chromosomal analysis. This detection rate increased to 5.2% when the pregnancy had a structural malformation on ultrasound [5, 7, 16–20, 23, 24]. In these studies the overall detection of array CGH over chromosomal analysis was 12%. When benign CNVs were removed and considered as normal results the detection rate dropped to 3.6% [24]; this percentage included the pathogenic CNVs as well as the variants of unknown significance (VOUS) with a potential of being pathogenic. The presence of VOUS was found in 1.1% of cases [24]. 

The ultrasound findings included cardiac abnormalities, increased nuchal translucencies, cystic hygromata or hydrops, or central nervous system abnormalities. Most of these studies used targeted BAC arrays [5, 16–20, 23], and some used both targeted and whole genome arrays [5, 16, 18]. The resolution for the arrays varied from 287 to 4685 BAC probes and from 44,000 to 946,000 oligonucleotide probes.

Tyreman et al. conducted a retrospective analysis of 106 karyotypically normal referrals with ultrasound findings using the GeneChip 6.0 SNP array from Affymetrix. This platform provides uniquely high resolution coverage of the genome with over 1.8 million probes, using oligonucleotide targets that provide copy number information only and single nucleotide polymorphisms (SNPs) oligonucleotide targets which provide genotyping as well as copy number information. In this study a total of 35 rare CNVs were identified, 10 (9%) of which were considered to be pathogenic, 12 were likely to be benign (11%), and 13 were VOUS (12%). The percentage of VOUS is slightly higher than the other studies because parental testing was not used in this study for their clarification. In addition in this study a case with a cryptic mosaic trisomy for chromosome 10 was identified as well as a case with loss of heterozygosity (LOH). The same platform can detect triploidy as well which is a major advantage; one of the limitations of array CGH is its inability to detect triploidies [7]. Table 3 shows the comparison between these studies.

In another study completed by Fiorentino et al. [21] pregnant women were referred for chromosomal and array CGH analyses. Both methods were carried out concurrently in order to compare results. A total of 1037 prenatal samples were studied, and the reason for referral of these samples included advanced maternal age, ultrasound findings, parental anxiety, and family history of a genetic condition or chromosome abnormality. Array CGH was carried out using whole genome BAC array with a resolution of 1 Mb across the genome and ~100 kb resolution in 139 regions associated with constitutional disorders. From the analysis it was determined that 13% of the samples had likely benign and of no clinical significance CNVs. Furthermore, array CGH revealed clinically significant chromosome alterations in 3.3% of the samples. In 0.9% of the samples array CGH provided diagnosis of clinically significant chromosomal abnormality which was not detected by chromosomal analysis and would have otherwise gone undetected. Clinically significant results were also identified by conventional cytogenetics as well in 73.5% of the total abnormalities also detected by array CGH (25/34) and in 2.4% of the total number of samples. 

Finally, in the largest prenatal study published to date by Wapner et al. which includes over 4000 cases, microarray analysis provided additional clinically relevant information in 1.7% of pregnancies with standard indications for prenatal diagnosis and in 6.0% of pregnancies with an anomaly on ultrasonography. In addition, uncertain findings (VOUS) occurred in 1.5% of all karyotypically normal cases. In total out of the 3822 normal karyotypes, 1234 common benign CNVs were identified (32.3%), 35 pathogenic CNVs (0.9%), and 130 VOUS (3.4%). Out of the 130 VOUS the 69 were likely to be benign, and the 61 were likely to be pathogenic. If we add the likely to be benign VOUS to the common CNVs, then the total of benign CNVs raises to 1303 (34.1%). If the likely to be pathogenic VOUS are added to the known pathogenic CNVs, then a total of 96 (2.5%) is reached. The authors do comment, however, that the number of VOUS is expected to fall, as additional experience is acquired. They also point out that for the interpretation of uncertain results, close collaboration between laboratory directors, clinical geneticists, counsellors and practitioners is necessary. This study also suggests that SNP arrays are used in prenatal testing to reliably identify triploidy which is missed with the use of standard arrays [22].

### 3.5. Can Array CGH Analysis Fully Replace Karyotyping?

Arrays CGH analysis is being introduced in prenatal diagnosis in conjunction to chromosomal analysis, but it cannot yet fully replace karyotyping for the following reasons: (a) it cannot detect balanced rearrangements such as translocations, balanced insertions, and inversions. This is especially important in Robertsonian translocations, as carriers of such are at high risk for uniparental disomy (UPD) [25] and the risks UPD imply. Even in the case were SNP arrays are used which can detect isodisomy [26], they cannot detect heterodisomy which is the most common form of UPD. In addition to Robertsonian translocations, balanced rearrangements especially *de novo *reciprocal translocations or insertions are important to be detected, as they can sometimes lead to abnormal phenotypes. Furthermore knowing the presence of a balanced rearrangement can provide the couple future risk assessments for an unbalanced offspring and information useful for reproductive planning, (b) it cannot detect low level mosaicism, a finding that we often see in prenatal diagnosis. Mosaicism is detected in 1-2% of CVS samples and in 0.2% of amniotic fluid samples [27]. Even though in about 84% of mosaic cases in CVS, the mosaicism is confined to the placenta [28], the remaining cases would have remained undetected if array CGH was the only method applied, and (c) it cannot always detect the presence of marker chromosomes, as was the case in one of our samples (Case 7), even in the nonmosaic state. Marker chromosomes are encountered in about 0.1% of prenatal diagnoses [27] and very often in the mosaic form. Depending on which chromosome they were derived from, their size, their inheritance mode and whether they are euchromatic or heterochromatic the phenotypic risk can be determined. In a study of 55 cases with marker chromosome it was demonstrated that out of the 26 nonmosaic markers only 14 were detected leaving 46% of array results normal. Even if this percentage reflects that the markers are mainly heterochromatic, the lack of detection does not completely exclude a possible phenotypic effect [29], and finally (d) it cannot visualize the type of rearrangement in the event where deletion or duplication detected by array CGH is proven to be *de novo* after parental testing [15].

### 3.6. Genetic Counselling

As genome-wide analysis is being introduced into prenatal diagnosis pretest counselling is of paramount importance due to the nature of the test and the findings emerging from the analysis. Information should be offered by counsellors, and everything should be explained clearly and in a nondirective way, so that prospective parents can make their own decision having their future child's best interest in mind.

It is imperative that the following information is given by the prospective parents:

- medical history of both parents;
- medical history of the pregnancy which should include any ultrasound findings;
- family pedigree of both parents up to three generations.

Counsellors should be aware of the state of mind parents-to-be are in, right after an ultrasound abnormality has been detected. Parents may not be able to absorb any information given to them at the time, so it is good practice to have everything written down as well, so that it is available for them to read later on. Following this, parental consent should be obtained. Prospective parents should be informed of the test, and its limitations should be further explained. They should know that the array technique cannot detect every single disease or well-known syndrome. In a study of 141 foetuses with ultrasound abnormalities and normal array results, there was a diagnosis in 15% of them when they were reviewed postnatally [30].

If, in the course of testing the foetus, whole genome array analysis is needed to be carried out for the parents, they should be counselled appropriately including informed consent on what information they want to receive.

The parents should be aware of all the possible outcomes of the array testing which could either be normal or abnormal. It should be explained to them that if CNVs are detected they could (a) explain the foetal ultrasound abnormalities, (b) be *de novo* and of unknown clinical significance, (c) be inherited and of unknown clinical significance, and (d) be an unsolicited finding unrelated to the ultrasound findings.

Variables of unknown significance and incidental findings are the most challenging for counsellors. This is why it is of prime importance to inform parents of such possible findings; an example is a late-onset inherited disease either *de novo* or inherited in the family. Its implications should be explained, and a distinction should be made between treatable (hereditary cancer) and nontreatable (Huntington's disease) late-on-set diseases. There is no straight forward guideline on how this should be carried out, but, for example, in Europe the current tendency is to ask parents whether they want to be informed about treatable late-onset diseases. Some laboratories even have a policy of not reporting unsolicited CNVs to nontreatable diseases [30]. There are many ethical questions arising from all these, one of them being the extent to which pregnant women and their partners should be allowed to determine the range of possible outcomes that will or will not be reported back to them [31]. National guidelines in the use of array CGH in prenatal diagnosis remain to be established.

## 4. Conclusions

Karyotyping has been the golden standard method for prenatal diagnosis for decades, being able to sufficiently diagnose numerical and large structural abnormalities (<3–10 Mb). With the introduction of array CGH analysis in postnatal analysis and its use as a first-tier test in cases of intellectual disabilities, it has been postulated that this method might someday actually replace conventional cytogenetics in prenatal diagnosis as well. Array CGH in a postnatal setting has been demonstrated to be a high throughput, comprehensive, and fast to detect copy number changes that can go undetected by light microscopy.

The current study has demonstrated that the usefulness of array CGH in prenatal diagnosis depends on the selection of the appropriate platform. More importantly, it has clearly shown that array CGH is a valuable tool in prenatal diagnosis, both in cases with foetal malformations and normal karyotype as well as in cases where an abnormality was detected with another method and further investigated with array CGH. Array CGH provided valuable information for phenotype-genotype correlation and provided more accurate information regarding the clinical significance and the risk in the current and future pregnancy of the respective patient. Another critical factor for accurate CNV classification is parental testing to determine between familial and *de novo* CNVs. Appropriate pre- and posttest genetic counsellings offer the prospective parents tools to decide on the management of their pregnancy. However, one of the problems posing dilemmas to genetic counsellors and something that array CGH has to overcome is the fact that it can detect coincidental findings, variants of unknown significance and variants with variable expressivity.

Currently the ideal setting to advance prenatal diagnosis and increase its resolution would be to apply array CGH in high risk pregnancies in conjunction with chromosomal analysis with a microarray designed especially for prenatal diagnosis. As we have seen, this increases the detection rate for likely pathogenic CNVs up to 5%. To avoid interpretation problems (previously discussed) these arrays should cover all known pathogenic CNVs and have a low-resolution backbone for the detection of relatively large CNVs thus keeping the detection of CNVs of unclear significance to the minimum. A shared database specifically dedicated to prenatal diagnosis coupled with the growing amount of data regarding CNVs and dosage sensitive genes could make it easier to interpret genomic arrays.

## Figures and Tables

**Table 1:** Subcategories of samples based on the reason for referral.

Category	Karyotype	Ultrasound findings	Number of samples
A	Normal	YES	53
B	Balanced rearrangement	YES	5
C	Balanced rearrangement	No	2
D	Abnormal	YES	1
E	Abnormal	No	4

**Table 2:** CNVs identified during array CGH analysis using CytoChip oligonucleotide arrays.

Case	Sample	GA	Reason for referral	Result	Status	Inh.	Clinical significance	Array type	Karyotype	Cat	Genome build
5	AF	17	Investigation of abnormal karyotype	mos 47,XY,+mar. arr 21q11.2q21.1(13,539,832-15,716,987)x3~4,21q21.3 (27,787,566-28,368,946)x3	Dup (2.1 Mb), Dup (0.5 Mb)	*De novo *	Significant	105 K	47,XY,+mar	E	NCBI36/hg18

9	CVS	13	Ultrasound abnormalities/hypoplastic nasal bone	arr 7q34q35(139,107,925-145,455,647x1)dn	Del (6.3 Mb)	*De novo *	Significant	105 K	46,XX,del(7)(q34q35)	D	NCBI36/hg18

12	AF	18	U/S Findings/NT thickness	arr 5p14.3p14.2(22,344,207-24,523,053)x3 pat,15q25.2q25.3(81,011,096-83,478,823)x1 dn	Del (2.4 Mb), Dup (2.2 Mb)	Pat, *de novo *	Significant	105 K	46,XY	A	NCBI36/hg18

29	CVS	12.2	Investigation of abnormal karyotype	47,XX,+mar/46,XX. arr 16p11.2p11.1(29,727,747-35,004,980)x2~3 dn	Dup (5.2 Mb)	*De novo *	Significant	105 K	47,XX,+mar/46,XX	E	NCBI36/hg18

31	AF	17	Investigation of abnormal results with MLPA	arr 22q11.21(17,274,865-19,891,492)x3 mat	Dup (2.6 Mb)	Mat	Significant	105 K	46,XY.mlpa 22q11.2(P023)x3 mat	E	NCBI36/hg18

34	CVS	13.2	U/S Findings	arr 9q34.3(139,754,208-141,102,496)x1 mat,arr 17p13.3(48,569-2,002,395)x3 mat	Del (1.35 Mb), Dup (1.95)	Mat	Significant	105 K	46,XY	A	GRCh37/hg19

36	CVS	12	U/S Findings/NT thickness	arr 7q31.1(112,763,119-113,252,118)x3 mat	Dup (0.5 Mb)	Mat	Unrelated to the RFR	180 K	46,XY	A	GRCh37/hg19

38	AF		U/S Findings/tetralogy of Fallot	arr 9q34.3(139,754,208-141,102,496)X3,17p13.3(48,569-2,002,395)X1 mat	Dup (1.35 Mb), Del (1.95)	Mat	Significant	180 K	46,XX	A	GRCh37/hg19

42	AF	25	U/S Findings/foetal anomaly, extremities artrogryposis	arr 10p15.3(1,011,902-1,396,788)x3 pat,15q21.1(49,491,651-49,809,467)x1 mat	Dup (0.38 Mb), Del (0.32 Mb)	Pat, mat	Unrelated to the RFR	180 K	46,XX	A	GRCh37/hg19

44	AF	21	U/S Findings/foetal abnormality, cardiac anomaly	arr 5q15(95,655,383-96,003,162)x1 pat	Del (0.35 Mb)	Pat	Unrelated to the RFR	180 K	46,XX	A	GRCh37/hg19

47	AF	16	U/S findings/increased NT= 3,7 mm	arr Xp22.33(716,598-1,224,238)x3 pat	Dup (0.5 Mb)	Pat	Unrelated to the RFR	180 K	46,XY	A	GRCh37/hg19

48	CVS		U/S Findings/small-asymmetric embryo	arr 7p22.2(4,137,938-4,677,493)x3 mat	Dup (0.53 Mb)	Mat	Unrelated to the RFR	180 K	46,XY	A	GRCh37/hg19

49	AF	23.2	U/S findings/aortic arch abnormality	arr Xp22.33(2,039,059-2,275,983)x3 mat	Dup (0.24 Mb)	Mat	Unrelated to the RFR	180 K	46,XY	A	GRCh37/hg19

65	AF	25.1	U/S findings/ ventriculomegaly	arr 4q35.1(185,787,238-186,132,543)x3 mat	Dup (0.35 Mb)	Mat	Unrelated to the RFR	105 K	46,XY	A	GRCh37/hg19
52	AF	20	U/S findings/bilateral hands polydactyly	Request family analysis before final report (array CGH), pending parental testing	VOUS	N/A	N/A	180 K	46,XX	A	GRCh37/hg19

61	AF	23.4	U/S findings/cataracts, limb abnormalities/IUD	Request family analysis before final report (array CGH), pending parental testing	VOUS	N/A	N/A	105 K	46,XX	A	GRCh37/hg19

63	AF	18	U/S findings/absence of nasal bone, hypoplastic	Request family analysis before final report (array CGH), pending parental testing	VOUS	N/A	N/A	105 K	46,XX	A	GRCh37/hg19

AF: amniotic fluid, CVS: chorionic villus sample, NT: nuchal translucency, IUGR: intrauterine growth retardation, Inh.: inheritance status, U/S findings: ultrasound findings, N/A: not applicable, GA: gestational age, Cat: category; Mat: maternal, Pat: paternal, RFR: reason for referral, VOUS: variable of unclear significance, and IUD: intrauterine death.

**Table 3:** Comparison between various studies which used array CGH in prenatal diagnosis.

Study	Array type	Karyotype/reason for referral	Results	Clinical significance of results
Kleeman et al., 2009 [16]	Signature prenatal targeted BAC chip V, signature whole genome chip	Normal karyotype/sonographic anomalies	4/50 abnormal	2% clinically significant, 6% inherited or benign variant

Vialard et al., 2009 [17]	Targeted genosensor BAC/PAC array	Normal karyotype/multiple congenital abnormalities	4/37 abnormal	10.8% clinically significant

Bi et al., 2008 [18]	BCM V6 oligonucleotide array	Normal karyotype/maternal age, sonographic anomalies, family history, and miscarriages	3/15 abnormal	13% clinically significant, 7% inherited or benign variant

Shaffer et al., 2008 [19]	Prenatal targeted BAC array	149/151 normal karyotype/maternal age, sonographic anomalies, family history, and parental anxiety	15/151 abnormal	1.3% clinically significant, 8% benign, and 0.5% unclear significance

Sahoo et al., 2006 [20]	BCM V4 targeted BAC array	93/98 normal karyotype/maternal age, sonographic anomalies, and family history	5/98 abnormal of which one had additional abnormalities	5% clinically significant

Tyreman et al., 2009 [7]	GeneChip SNP whole genome oligonucleotide array	Sonogramphic abnormalities	35/106 abnormal	9% likely pathogenic, 12% likely benign, and 13% unclear significance

Coppinger et al., 2009 [5]	Signature V 4.0, prenatal targeted BAC array, and whole genome array	Normal karyotype/maternal age, sonographic anomalies, family history, and anxiety	*Whole genome*: 22/180 abnormal *Targeted*: 7/62 abnormal	*Whole genome*: 2.7% clinically significant, 0.5% unclear significance, and 8.8% benign variants *Targeted*: 0.9% clinically significant, 0.5 unclear significance, and 8% benign variants

Fiorentino et al., 2011 [21]	Whole genome CytoChip focus BAC array	Maternal age, sonographic anomalies, family history, and anxiety	34/1037 abnormal	3.3% clinically significant, 13% benign variants.

Wapner et al., 2012 [22]	Agilent 44 K targeted array Affymetrix Genome-Wide Human SNP Array 6.0	Normal karyotype/maternal age, sonographic anomalies, abnormal serum biochemistry, family history, anxiety, and previous pregnancy with abnormality	1399/3822 (36.6%)	2.5% pathogenic and likely to be pathogenic, 32.3% common benign CNVs (34.1% if the likely to be benign VOUS is added), and 3.4% unclear significance (1.8% likely to be benign and 1.6% potential for clinical significance)

Our study	Whole genome105K or 180 K CytoChip oligo arrays	Normal karyotype and sonographic anomalies, balanced rearrangements with or without sonographic anomalies, abnormal karyotype, or MLPA	17/64 abnormal	4.7%clinically significant, 10.9% inherited or benign variants, and 4.6% unclear significance
